# Evaluating the efficacy of inhaled amikacin as an adjunct to intravenous combination therapy (ceftazidime and amikacin) in pediatric cystic fibrosis pulmonary exacerbation

**DOI:** 10.3389/fphar.2023.1130374

**Published:** 2023-03-09

**Authors:** Amin Rakhshan, Nazanin Farahbakhsh, Ghamartaj Khanbabaee, Seyed Ahmad Tabatabaii, Saeed Sadr, Maryam Hassanzad, Mohammad Sistanizad, Farzaneh Dastan, Mahmoud Hajipour, Amir Reza Bahadori, Bahador Mirrahimi

**Affiliations:** ^1^ Department of Clinical Pharmacy, School of Pharmacy, Shahid Beheshti University of Medical Sciences, Tehran, Iran; ^2^ Department of Pediatric Pulmonology, Mofid Children’s Hospital, Shahid Beheshti University of Medical Sciences, Tehran, Iran; ^3^ Pediatric Respiratory Diseases Research Center, National Research Institute of Tuberculosis and Lung Diseases, Shahid Beheshti University of Medical Sciences, Tehran, Iran; ^4^ Children’s Gastroenterology, Liver and Nutrition Research Center, Shahid Beheshti University of Medical Sciences, Tehran, Iran; ^5^ Student Research Committee, Shiraz University of Medical Sciences, Shiraz, Iran

**Keywords:** aminoglycosides, cystic fibrosis-CF, nebulizer, psedudomonas aeruginosa, toxicicity

## Abstract

**Background:** *Pseudomonas aeruginosa* is the most common microorganism found in the sputum culture of Cystic fibrosis (CF) patients causing the pulmonary destruction. Aminoglycosides have a low diffuse rate from lipid membranes, and respiratory system secretions. Regarding the burden of pulmonary exacerbation caused by the *pseudomonas aeruginosa* in cystic fibrosis patients in the long term and the limited number of clinical trials focused on appropriate treatment strategies, the present study evaluated the concurrent inhaled and intravenous aminoglycoside antibiotics for pulmonary exacerbation caused by the *pseudomonas aeruginosa* as a safe and effective treatment in children.

**Method:** This study was a blinded, randomized clinical trial phase conducted in a tertiary referral pediatric teaching hospital from May 2021 to May 2022. The patients were randomly allocated to receive intravenously administered ceftazidime and Amikacin alone or with inhaled Amikacin. Forced expiratory volume (FEV1), Amikacin *via* the level, kidney function tests, audiometry, inflammatory markers (erythrocyte sedimentation rate and C-reactive protein), hospital stay, and bacterial eradication rate were compared in two therapy groups.

**Results:** the average FEV_1_ has increased by 47% in Neb + group compared to Neb− group following treatment. Hospital stay was lower in Neb + group. No renal toxicity or ototoxicity was observed in both therapy groups. *Pseudomonas aeruginosa* eradication rate Neb− and Neb + groups were 44% and 69%, respectively (*p*-value = 0.15).

**Conclusion:** Concurrent inhaled and intravenous Amikacin is safe and effective to treat *Pseudomonas aeruginosa* exacerbation in CF patients. Moreover, co-delivery antibiotics’ route treatment increased the eradication rate. Although not statistically significant, never the less, it is clinically relevant. The intervention reduced the length of hospitalization in this group.

**Clinical Trial Registration:** clinicaltrials.gov, identifier [IRCT20120415009475N10].

## 1 Introduction

Cystic fibrosis (CF) is a hereditary illness involving various human systems, especially respiratory system. CF is an autosomal recessive disease caused by a defect in the CFTR gene located in the 7q31.2 chromosome, producing cystic fibrosis transmembrane conductance regulator (CFTR protein). Because it affects ion channels, CFTR protein disruption plays a crucial part in the pathophysiology of the trans-membranous epithelium of the pulmonary, sweat gland, digestive, and reproductive issues in CF patients. CF patients cannot appropriately secret salt and water across epithelia resulting in the viscous, thickened secretions. Therefore, maladapted airway clearance induced bacterial colonization and inflammatory responses, leading to the pulmonary exacerbation (Radlović, 2012). Among various CF respiratory complications, many patients present problems, such as cough, bronchiolitis, pulmonary atelectasis, and pneumonia. Considering the lower effectivity of the innate immune system of the patient with CF in respiratory systems compared to others, infections in this system are more common by microorganisms, such as *Staphylococcus aureus*, *Pseudomonas aeruginosa*, and Burkholderia cepacia (Blanchard and Waters, 2019).

Since *Pseudomonas aeruginosa* is the most prevalent bacteria isolated from the sputum of cystic fibrosis (CF) patients and is responsible for the deterioration in pulmonary function, it is crucial to choose the correct antibiotic therapy in order to eradicate this pathogen. (Taccetti et al., 2012). According to different studies, double antibiotics treatment should be selected for the coverage of *Pseudomonas aeruginosa*; primarily, beta-lactam and aminoglycoside combination is recommended for their synergistic effect (Flume et al., 2009; Ratjen et al., 2009). Despite the marketing of newer antibiotics, aminoglycoside antibiotics are still widely used as an effective treatment for the cystic fibrosis pulmonary exacerbations against *Pseudomonas aeruginosa* (Flume et al., 2009; den Hollander et al., 1997). Aminoglycoside antibiotics are effective against numerous Gram-negative aerobic bacilli by irreversibly binding to 30 s subunit of ribosomes, causing codon reading errors and leading to cell death (Young et al., 2013). Considering the renal toxicity may occur when aminoglycoside antibiotics are taken, most guidelines recommend that a once-daily dose is preferred to divided dosages (Flume et al., 2009). Although using two different drug delivery routes may increase antimicrobial effects by helping improve drug delivery to the respiratory tract, an increasing risk of aminoglycoside side effects is expected. Therefore, the pharmacokinetic studies and therapeutic drug monitoring while using inhalation and intravenous medication can help achieve the most effective treatment regime for microbial eradication and reduce the risk of side effects. Thus, based on CFF (Cystic Fibrosis Foundation) recommendation, the decision to use inhaled aminoglycoside concomitantly with intravenous aminoglycoside should be individually made for each patient until further studies are conducted in this regard (Flume et al., 2009; Ratjen et al., 2009; Hewer et al., 2020).

Several studies showed that dual antibiotic therapy is required to treat *Pseudomonas aeruginosa*. The systemic administration of aminoglycosides alone is not particularly effective to eradicate CF exacerbation phase in terms of low diffuse rate of aminoglycosides from the lipid membrane to respiratory system secretion. For a reason mentioned above, some studies recommended adding inhaled antibiotics to increase the aminoglycoside concentration in CF patients’ respiratory tracts and bronchial tract (Langton Hewer and Smyth, 2017; Blanchard and Waters, 2019; Taccetti et al., 2021).

Considering the burden of pulmonary exacerbation caused by *pseudomonas aeruginosa* in cystic fibrosis patients in long term and the limited number of clinical trials focused on appropriate treatment strategies, the present study evaluated the concurrent inhaled, and intravenous aminoglycoside antibiotics for the pulmonary exacerbation caused by *pseudomonas aeruginosa* as a safe and effective treatment in children.

## 2 Materials and Method

### 2.1 Study design and patients

This study was a blinded randomized clinical trial phase three conducted in a tertiary referral pediatric teaching hospital from May 2021 to May 2022. Inclusion criteria were patients with CF admitted in terms of CF exacerbation to the pulmonary ward at Mofid children’s Hospital, Shahid Beheshti University of medical science. The patients should be between 6–18 years old and have a sputum culture positive with *pseudomonas aeruginosa*. A pulmonary exacerbation was described by the Cystic Fibrosis Foundation as having an increased cough, more sputum being produced, shortness of breath, chest discomfort, reduced appetite, weight loss, and decreased pulmonary function tests (Flume et al., 2009). Patients who had a history of allergy to aminoglycosides or beta-lactam antibiotics, chronic renal failure from stage 3 to end-stage stage according to KDIGO (Kidney Disease Improving Global Outcomes) guidelines, Liver failure (acute or chronic) defined as Child-Pugh class B or C or a liver enzyme test above five times the normal upper limit, congenital metabolic disorders, myopathies, hearing impairment (>15 dB hearing level at a *range* of 125 *Hz*–6000 *Hz* on standard Audiogram), neuromuscular diseases (such as Amyotrophic lateral sclerosis (ALS), Cramp-fasciculation syndrome, *etc.*), severe electrolyte imbalance (including potassium less than 2.5 or more Than 5.5; Sodium less than 125 or more than 155; Magnesium less than 1.8 or more than 2.2) and had a positive COVID-19 Polymerase Chain Reaction (PCR) at admission were excluded.

University Ethics Committee approved the trial (with code IR. SBMU.PHARMACY.REC.1400.039), and informed consent was obtained from patients or their legal guardians. The trial was registered at Iranian registry of clinical trials by the code IRCT20120415009475N10. The sample size was estimated with a confidence level of 95 percent and power of 80 percent with data from previous studies of 40 Patients divided into two main groups of 20; Patients in Neb− Group received intravenous Ceftazidime, intravenous Amikacin and inhaled NaCl 0.9%. Patients in the Neb + group received intravenous Ceftazidime, intravenous Amikacin, and inhaled Amikacin.

Amikacin 250 mg (for patients under 50 kg) or 500 mg (for patients over 50 kg) were added to 3 ccs 0.9% sodium chloride and was instilled into a vibrating mesh nebulizer chamber for use. The nebulizer used was a vibrating mesh nebulizer, PARI eFlow®rapid. All patients utilized the identical kind of device. 5 ml of NaCl 0.9% was utilized as a placebo in the nebulizer chamber for participants in the Neb-group. Patients in both groups received standard-of-practice care according to Cystic Fibrosis Foundation Guideline (Flume et al., 2009), including an intravenous infusion of Ceftazidime (250 mg/kg/day divided every 8 h) and Amikacin (25 mg/kg/day every 24 h), a set anti-pseudomonas combination. Other supportive treatments, including chest physiotherapy, adequate hydration, supplemental oxygen, oral pancreatic enzymes supplement, strict COVID-19 isolation, and a high-calorie diet, were considered equally for the patients in both groups.

### 2.2 Randomization

Block Randomization method was used for the randomization of samples. The patients were allocated based on quadruple blocks using the sealedenvelope.com website. The encoded normal saline *versus* amikacin-containing syringes were prepared in identical packaging, and non-sequential alphanumerical codes provided by sealedenvelope.com were used for blinding. The patient, caregiver, researcher, and statistical analyzer were blinded.

### 2.3 Clinical outcome parameters

After recording the demographic information for each patient on admission, the medical staff requested a complete laboratory profile. Spirometry was performed on days 0 and 14^th^ to evaluate baseline and final FEV_1_. Changes in the FEV_1_ parameter were considered the main finding and as the primary outcome to determine the effectiveness of treatment. The renal function was calculated every other day by eGFR was calculated according to the revised Schwartz formula as follows: eGFR (ml/min × 1.73 m^2^) = 0.413 × Ht (cm)/SCreat (mg/dl). Liver function tests (aminotransferases, bilirubin, INR), Albumin level, and inflammatory parameters (ESR (erythrocyte sedimentation rate) and CRP (C-reactive protein)) were assessed at the baseline, 7th day, and 14th day of treatment. O_2_ saturation, Temperature, and Respiratory rate were measured and recorded daily. The hospital stays for each patient and the need for intensive care unit (ICU) admission -if needed-were recorded.

### 2.4 Audiometry

All patients had pure tone auditory testing at baseline and after the conclusion of therapy. This test is performed by presenting a pure sound to the ear through the earphone and measuring the lowest intensity in decibels (dB), and the standard Audiogram was used to measure hearing levels between 125 Hz and 6000 Hz. Results of the audiometry tests were provided in both quantitative and qualitative ways.

### 2.5 Microbiology

Microbiological cultures and antibiotic susceptibility testing were performed on sputum samples at baseline and the end of the treatment (day 14^th^) for all patients. Sputum samples were taken from patients with the help of bronchoalveolar lavage and transferred to the laboratory under appropriate conditions for examination and culture.

### 2.6 Pharmacokinetic assessment

Blood samples were collected from all patients on 4^th^ day of treatment 30 min before receiving the 4^th^ dose of Amikacin. The blood samples were collected in the anticoagulated tubes. The serum of blood samples was separated by centrifuging at 1100–1300 rpm for 15 min. The amikacin trough level was measured with Roche COBAS INTEGRA 400 (Produced in Roche Diagnostics GmbH Company Mannheim, Germany) access auto analyzer by fluorescence polarization immunoassay (FPIA) test.

### 2.7 Statistical analysis

SPSS statistical software was used for statistical analysis. First, by defining the variables, the data were entered into SPSS software, and then the descriptive results of the quantitative variables were reported.

All analysis were performed based on the same pattern of initial randomization of patients and intention-to-treat (ITT).

Multiple linear regression modeling was used to adjust confounding variables.

## 3 Results

### 3.1 Study population

**FIGURE 1:**
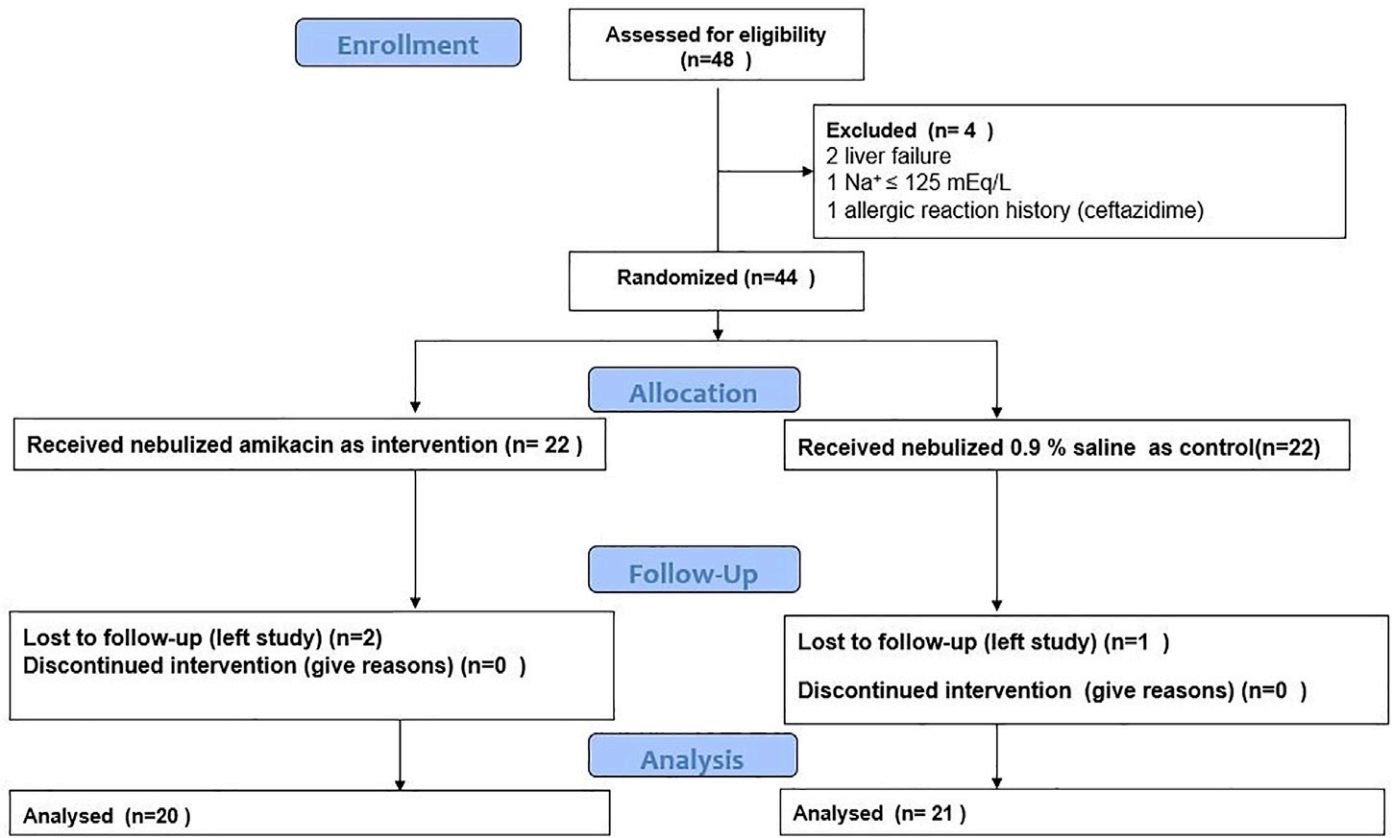
Study CONSORT diagram.

The summary of process carried out in the study is shown in Figure 1. Patients’ baseline characteristics are shown in Table 1. There was no significant difference between two groups regarding age, sex, ESR, CRP, and pulmonary functions at baseline. Two patients from Neb+ and one from Neb-groups were lost to follow-up because they left in terms of the hospital’s strict COVID-19 isolation policy and continued treatment in other centers.

**TABLE 1:** Baseline characteristics of the study patients

	Total	Neb− group	Neb+ group	*p* value
Age (y)	13.61±4.13	13.73±3.49	13.50±4.69	N.S
Number of patients	41	21	20	
Male (%)	17(41.5%)	5(23.9%)	12(60.0%)	N.S
Female (%)	24(58.5%)	16(76.1%)	8(40.0%)	N.S
Pulmonary function FEV_1_ (L)	1.18±0.62	0.98±0.47	1.31±0.66	N.S
ESR (mm/h)	36.3±29.9	42.52±33.14	26.95±19.43	N.S
CRP (mg/L)	22.4±17.0	24.79±17.81	25.17±17.71	N.S

Abbreviations: FEV1: Forced expiratory volume in second 1; N.S: Not significant; ESR: erythrocyte sedimentation rate; CRP: C reactive protein.

### 3.2 Clinical outcome

The changes in clinical outcome for Neb- and Neb + groups are shown in Table 2.

**TABLE 2:** Changes in lung function and inflammatory markers after 14 days of treatment in Neb+ group vs. Neb- group.

Parameters:	Neb− group			Neb+ group		
	**Before**	**After**	***p* value**	**Before**	**After**	***p* value**
FEV_1_(L)	0.98±0.47	1.14±0.60	*<*0.05	1.31±0.66	1.67±0.71	< 0.001
ESR (mm/h)	42.52±33.14	26.95±21.18	< 0.001	26.95±19.43	9.50±10.33	< 0.001
CRP (mg/L)	24.79±17.81	7.58±6.88	< 0.001	25.17±17.71	5.61±4.14	< 0.001

Abbreviations: FEV1: Forced expiratory volume in second 1; ESR: erythrocyte sedimentation rate; CRP: C reactive protein.

FEV_1_ at the baseline and end of the treatment was compared in two groups by paired sample t-test. There was a significant difference between baseline and final FEV_1_ (*p*-value <0.001 for Neb + group and *p*-value = 0.02 for Neb-group) in both groups. The differences in the FEV_1_ at baseline and the end of the treatment were compared between two groups. In the Neb-group, the average FEV_1_ at baseline was 0.98 L; at the end of treatment, it was 1.14 L, which increased by 0.16 L. In contrast, in the Neb + group, the average FEV_1_ at baseline was 1.31 L and at the end of treatment was 1.67 L which had an increase of 0.36 L. The coefficient of determination (R^2^), according to linear regression modeling was 52% (R squared = 0.52) and The average FEV1 increased by 0.47 in the Neb + group compared to the Neb-group after therapy (ß = 0.47). The improvement in FEV1 level is significantly correlated with getting additional amikacin nebulizer therapy, according to this research. The changes in the level of inflammatory markers (ESR and CRP) are shown in Table 2. Using the openepi.com website, considering the mean and standard deviation of the FEV1 level at the end of the study, the power of the study was higher than 80%.

**FIGURE 2:**
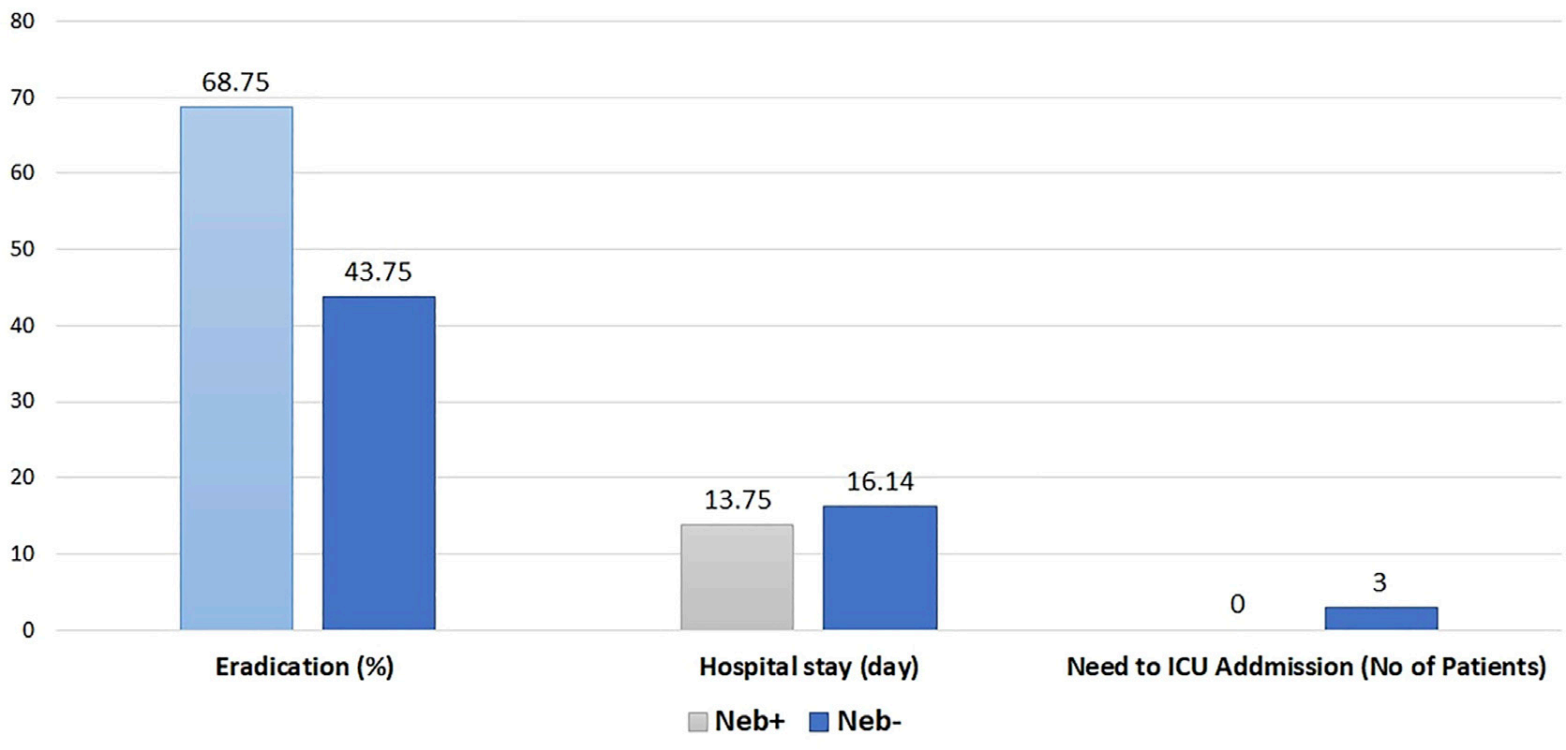
Rate of eradication (*p* value = 0.15), Hospital stay (*p* value = 0.3), Need to ICU admission (*p* value < 0.05).

Microbial outcome (eradication rate), length of hospital stay, and need for ICU admission in both groups are shown in Figure 2.

### 3.3 Adverse effects

The risk of hearing loss as an adverse effect associated with the aminoglycoside antibiotics was assessed by pure tone audiometry at the baseline and end of the treatment. Based on quantitative data from Audiogram, covering frequencies between 125 and 6000 Hz, none of the patients had a qualitative decline in hearing level, and all patients had normal hearing at the conclusion of treatment. Kidney function was monitored every other day by measuring the serum creatinine and calculating the Schwartz Formula equation. None of the patients developed renal impairment during the study period. Electrolyte levels of the patients were in the normal range during the study.

### 3.4 Microbiological outcome

At baseline, per inclusion criteria, *Pseudomonas aeruginosa* was confirmed in all Patients’ sputum samples. McNemar’s and chi-square test was performed to check the difference in the eradication rate between the two groups in the analysis. In the Neb + group, at the end of treatment, eradication occurred in 68.75%; While in the Neb-group, eradication occurred in 43.75% of patients. These results are shown in Figure 2.

### 3.5 Amikacin serum concentrations

Amikacin trough level for all patients before receiving the fourth dose was measured. The average trough in Neb- and Neb + groups were 2.01 and 1.61, respectively. There was no significant difference in the amikacin trough level between the two groups.

## 4 Discussion

The present randomized clinical trial compares two treatments for *Pseudomonas aeruginosa* exacerbation in CF patients, between intravenous Amikacin and Ceftazidime and adding inhaled Amikacin to the same treatment. Our investigation proved the benefit of supplementing the conventional therapy with inhaled Amikacin. This research is interesting since it was conducted during the exacerbation phase, whereas most prior studies were conducted on stable CF patients. This study was designed to address the concern for high aminoglycoside plasma levels regarding the concomitant use of inhaled and intravenous aminoglycoside. The ototoxicity and renal toxicity related to aminoglycoside accumulation were explicitly monitored. There was no significant difference among plasma levels in the two groups, and no ototoxicity or renal toxicity was monitored.

FEV_1_ is one of the best objective parameters to monitor lung function in CF patients. By calculating the amount of air forced in the first second of breathing time from the patient’s lungs, FEV_1_ was measured as a ratio (Taccetti et al., 2012). The difference between FEV_1_ in both groups was none significant initially. We collected final FEV_1_ at the end of the study (after exacerbation recovered) to compare the effect of the intervention. Even though both groups’ final FEV1 levels increased just slightly, the Neb + group, which got supplemental inhaled Amikacin, had a much higher FEV1 percentage than the control group. Schaad et al. conducted a similar study on 87 CF patients during the exacerbation phase and demonstrated pulmonary lung function by plethysmography and not by spirometry; therefore, the parameters they measured were forced vital capacity and airway resistance, which improved after treatment. They did not show significant improvement in lung function in patients who received additional inhaled Amikacin (Schaad et al., 1987).

ELITE study and other similar studies showed that inhaled aminoglycosides, such as tobramycin or gentamicin (it has the same effect as Amikacin in Gram-negative germs) were effective in long-term eradication of *Pseudomonas* (Schaad et al., 1987; Ratjen et al., 2010; Taccetti et al., 2012; Van Stormbroek et al., 2019). In line with the ELITE trial, but at the exacerbation stage, the current study demonstrated the effects of inhaled Amikacin in CF patients. The EPIC study, on the other hand, found no discernible differences between IV and inhaled antibiotic therapy as compared to IV administration alone (Treggiari et al., 2011). In Schaad et al. study, the eradication rate of *Pseudomonas* was 41% and 70% in Neb- and Neb + groups, comparable to our study (Schaad et al., 1987).

It is perceived that the pharmacokinetics of antibiotics in the CF patients differ from others. It is essential to conduct pharmacokinetic studies to maximize clinical effects, select optimal treatment regimens, and minimize the side effects of antibiotics (Caceres Guido et al., 2019). When using Amikacin, ototoxicity and renal toxicity are possible side effects (Mulheran et al., 2001; Young et al., 2013). When using aminoglycosides, a daily dose rather than many doses per day is advised to lower the risk of renal problems. Although the concurrent inhalation and IV antibiotic delivery routes can increase the aminoglycoside effectiveness, they may increase the possibility of systemic side effects (Akkerman-Nijland et al., 2021). It ought to be noted that studies in children showed that, generally, aminoglycosides are cleared from the blood faster than adults. Compared to the adults and normal populations, the pharmacokinetic parameters in children and CF patients are fundamentally different, which justifies the need for independent studies on this particular group of patients (Caceres Guido et al., 2019).

Both groups were screened for ototoxicity and renal toxicity, and no one appeared with these adverse effects. A similar result was seen in Schaad et al. study (Schaad et al., 1987). We compared the drug toxicity of both groups by measuring the serum trough levels, and no significant differences between the groups were observed. These data show adding inhaled aminoglycosides to standard treatment does not have substantial systemic absorption or side effects. In other words, both groups of our trial have the same plasma levels, but Neb + group that received concurrent IV and inhaled Amikacin had better clinical outcomes with no extra side effects.

Strombroek et al. study describes that co-treatment with nebulized and IV routes of antibiotics *versus* only the IV route has reduced the length of hospitalization, and it is appropriate to use both instead of just the IV route. Therefore, by spending less on treating CF patients, this strategy has helped nations’ health systems by lowering overall treatment costs (Van Stormbroek et al., 2019). Patients in this trial who received IV and inhalation antibiotics were released sooner than those in the control group. The cost-benefit of earlier discharge may be interesting to study the reduction in both the marginal and opportunity cost of disease.

*Pseudomonas* colonization creates a significant disease burden for CF patients in the long term. The residing bacteria, directly and indirectly, destroys lung function and any intervention to eradicate *pseudomonas* infection is crucial for the patients, even though the infection would return in time. The eradication rate was not significantly different among groups, but it was noticeably higher in Neb + group; this finding is valuable for clinical practice and helpful in designing future studies.

## 5 Conclusion

Concurrent inhaled, intravenous Amikacin is safe and effective to treat the *Pseudomonas aeruginosa* exacerbation in CF patients. Moreover, co-delivery antibiotics’ route treatment increased the eradication rate. Although not statistically significant, never the less, it is clinically meaningful. The intervention reduced the length of hospitalization in this group.

## Data Availability

The raw data supporting the conclusion of this article will be made available by the authors, without undue reservation.
